# High correlation of the proteome patterns in bone marrow and peripheral blood blast cells in patients with acute myeloid leukemia

**DOI:** 10.1186/1479-5876-7-7

**Published:** 2009-01-15

**Authors:** Gero Hütter, Anne Letsch, Daniel Nowak, Julia Poland, Pranav Sinha, Eckhard Thiel, Wolf-K Hofmann

**Affiliations:** 1 Department of Internal Medicine III (Hematology, Onkology), Charité Berlin Campus Benjamin Franklin, Berlin, Germany; 2 Institute of Laboratory Medicine and Clinical Chemistry, LKH Klagenfurt, Austria

## Abstract

**Background:**

When comparing myelogenous blasts from bone marrow and peripheral blood, immunophenotyping usually show a strong correlation of expression of surface antigens. However, it remains to be determined, whether this correlation also exists on the level of protein expression.

**Method:**

Therefore, we investigated both bone marrow and peripheral blood blast cells from six patients with newly diagnosed acute myeloid leukemia (AML) using conventional two-dimensional electrophoresis in the first dimension and linear polyacrylamide gels (12%) in the second dimension. Proteins were visualized using the silver staining method and image analysis was performed using the PDQuest system.

**Results:**

For each patient over 80 proteins were evaluated in the sample from peripheral blood and bone marrow. We could demonstrate that the protein expression profile of bone marrow did not significantly differ from the expression patterns of peripheral blast cells.

**Conclusion:**

The proteome-set of leukemic blast cells from marrow and blood, does not differ substantially when drawn from AML patients with over 80 percent blast cells in both compartments. This indicates that in AML, blasts from peripheral blood samples can be considered suitable for investigations of the proteome using 2D-electrophoresis.

## Background

Acute myeloid leukemia (AML) is an aggressive hematological neoplasia and it is characterized by accumulating myeloid precursor cells in bone marrow and detection of such cells in peripheral blood. Cytogenetics and molecular diagnostics are helpful for an individualized therapy in certain subsets of AML. There is hope that proteomics in AML will prompt new diagnostic or therapeutic biomarkers in future [1]. Up to date, the contribution of proteomics to the management of patients with AML is negligible although an enormous effort has been undertaken to develop databases of cancer proteins detected by two-dimensional gel electrophoresis [2]. They contain 2-D patterns and information from patients with lymphoproliferative disorders, leukemia, and a variety of other cell populations [3-6]. These databases were developed primarily from *in vitro *cell cultures. Experiences with corresponding *in vivo *samples are rare, even though cells from hematological disorders can easily be obtained for protein analysis. First investigations referring to the proteome of leukemia *in vivo *were undertaken from Hanash in the middle 80's. Hanash screened polypeptides as markers to distinguish acute lymphoblastic leukemia (ALL) cell lineages [7]. Later the proteomic approach was used to identify Hsp27, which distinguishes between ALL in infants and older children [8,9]. Recently, Balkhi and co-workers were able to identify significant differences in the AML proteome between cytogenetic groups of this disease. They postulated, that analysis of the post-translational modifications could be useful to distinguish different subgroups of AML [10].

Studies employing immunophenotyping methods in acute myeloid leukemias (AML) have shown a strong correlation of surface antigen expression comparing bone marrow and peripheral blood blast cells [11]. However, it remains unclear, whether there are differences in expression levels on either protein or RNA-level which may indicate biological differences for both cell types.

In the present study, we aimed to investigate the profile of protein expression of blast populations from peripheral blood and bone marrow aspirates using a proteomic approach with 2D-electrophoresis in newly diagnosed patients with AML.

## Materials and methods

### Sample preparation and solubilization

Blast samples from bone marrow aspirates and peripheral blood were isolated from six patients with Ficoll-centrifugation and washed at least three times in large volumes of phosphate-buffered saline (Table 1). The cell pellet was solubilized according to Rabilloud in 9 M urea, 4% w/v CHAPS, and 20 mM spermine and 40 mM DTT [12]. After centrifugation to remove the precipitated nucleic acids, the supernatant was collected, for protein determination and for proteomic analysis.

**Table 1:** Patient and sample characteristics.

*Patient*	*Sample-ID*	*Age*	*Gender*	*FAB-subtype*	*Karyotype*	*Source*	*WBC in μL (% blasts)*
A	#02-05	60	Female	M2	t(8;21)	PB	4.8 (80%)
	#02-02					BM	*
B	#02-06	22	Female	M2	normal	PB	379.0 (93%)
	#02-03					BM	*
C	#02-24	63	Female	M5b	normal	PB	120.0 (91%)
	#02-25					BM	*
D	#02-33	46	Male	M1	complex	PB	11.2 (85%)
	#02-34					BM	*
E	#02-37	27	Female	M0	t(9;11)	PB	5.2 (81%)
	#02-36					BM	*
F	#02-39	58	Male	M4	normal	PB	37.0 (87%)
	#02-38					BM	*

WBC = white blood cell count, * = bone marrow samples were adjusted to 1.000.000 mononuclear cells per assay.

### Protein determination

Since high concentration of urea and detergents interfere with any available protein assay system, we adapted a turbidimetric assay especially for samples prepared for 2D analysis[13]. In this assay, proteins are precipitated by trichloroacetic acid and measured turbidimetrically at 720 nm. Briefly, 35 ml of each sample was pipetted in duplicate in wells of a 96-well microtitre plates (Nunc, Denmark). One hundred ml of 0.1 M HCl was added to each well and the mixture shaken for 1 mm. Twenty five ml of 20% TCA was added to each well. The optical density was measured at 720 nm 5 min after TCA-addition using a standard Dynatech MR 7000 ELISA photometer (Dynatech, Hamburg). For evaluation, a non-linear standard curve with protein concentrations of 0.2, 1, 2 and 5 mg/ml was used. Control material from Boehringer Mannheim (Precinorm protein control serum) was used to obtain the standard curves that were run with each determination.

### First dimension isoelectric focusing (IEF)

First dimension glass tubes were placed in the Hoefer cast-system. Solution for IEF contains 8.24 g urea, 1.95 ml acrylamide solution (T = 28.38%, C = 1.92%)^1^, 600 μl carrier-ampholyte (CA) 5–7 (Servalyt), 200 μl CA 3.5–10 (Pharmacia), 3 ml Triton X 10%, 20 μl TEMED, and 30 μl ammonium persulfate 10%. The cathodic chamber was filled with 10 mM of sodium hydroxide and the anodic chamber with 3.26 ml phosphoric acid 85%. The solution for the overlay contained 20% glycerol and 2% CA. Focusing started with 200 V for 15 minutes, followed by 300 V for 30 minutes and finally with 400 V for 60 minutes. After IE-focusing, the sample was added on the cathodic side of the tube gel. The aliquot of the sample contained a total of 10 μg of protein. Electrophoresis started with 200 V for 15 minutes, followed by 300 V for 30 minutes and finally 400 V for 12 hours.

### Second dimension SDS-page

Tube-gels were sealed on top of linear polyacrylamide gels (T = 30%, C = 2%) using a sealing solution (1% agarose, 0.2% SDS, 0.15 M Bis/Tris, 0.1 M HCl). The Iso-Dalt System contained a buffer of 58 g tris base, 299 g glycine, and 100 ml SDS 20%. The run was completed at 20 mA/gel until the tracking dye reached the bottom of the gel [14]. After electrophoresis, the gels were fixed in 50% ethanol, and 10% acetic acid for 12 hours.

### Silver staining

Proteins were visualized using the silver staining method employing a modification of the method of Heukeshoven according to Sinha et al. [15,16].

### Image Analysis and Spot Identification

Image analysis was performed using the PDQuest system according to the protocols provided by the manufacturer after scanning with the densitometer GS-710 (Bio-Rad, CA, USA), the spot pattern of each patient sample was summarized in a gel image. For protein identification, each gel image was matched to the previously 130 identified spots of the gastric carcinoma cell line EPG85-257 [17]. To yield information about changes in the protein expression gel images of peripheral and blood sample for each patient were matched. The following criteria for differential protein expression were used: (i) spot intensity: four-fold increased = differential overexpression; (ii) spot intensity: four-fold decreased = differential under-expression.

## Results

### Matching of samples

**Figure 1:**
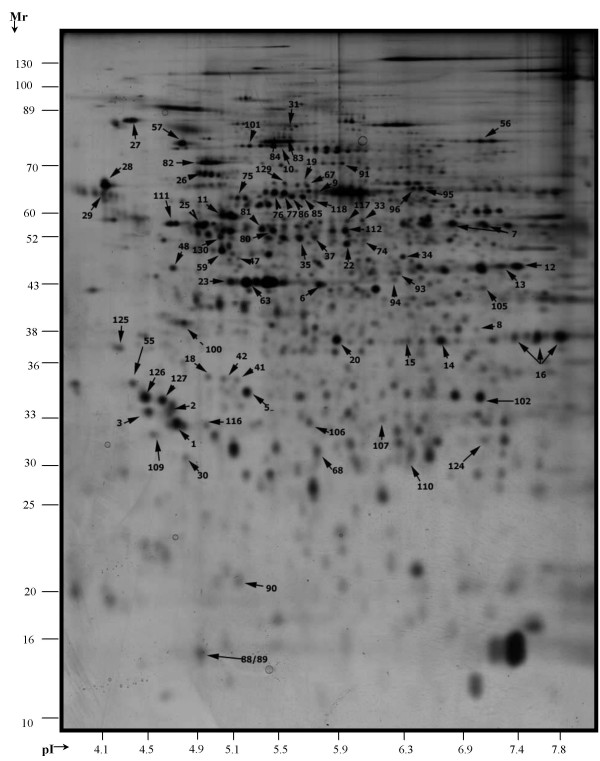
**2-D pattern of the silver stained gel image of the master gel image**. 2-D pattern of the silver stained gel image of a master gel image containing the spot information of all investigated samples. For protein identification, each gel image was matched to the previously 130 identified spots of the gastric carcinoma cell line EPG85-257. Proteins identified to date are marked with arrows and numbered according to Sinha et al. [17].

In the pH range 4.0–8.0, conventional 2-D electrophoresis of the 12 samples yielded about 700–900 spots, respectively (Figure 1). We were able to identify a maximum of 107 proteins in the AML samples. 23 Spots of the gastric cancer cell line were not represented in the AML samples.

**Figure 2:**
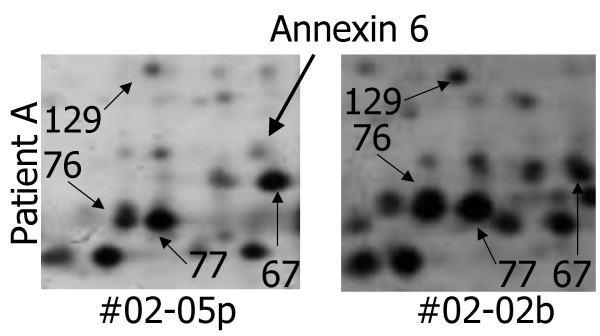
**Detail of the two-dimensional patterns of patient A**. Detail of the two-dimensional patterns of patient A. Different expression of spot 19 (annexin 6) in the bone marrow sample in comparison to peripheral blood.

Intra-individual analysis of the spot patterns showed a high correlation between the sample from peripheral blood and bone marrow (Table 2). On/off-phenomena of the identified spots were observed in four cases: Spot No. 19 (annexin 6) was found in patient A in the sample of peripheral blood but not in bone marrow, in patient B an inverse constellation was detected concerning this protein (Figure 2). As a third variance an absence of spot No. 102 (phosphoglyceromutase) was only found in the bone marrow of patient B. The fourth change concerned spot No. 130 (vimentin) which was only represented in the peripheral blood sample of patient B.

**Table 2:** Different protein expression in AML.

	Patient/Sample-No.	A		B		C		D		E		F	
		
Spot-No.	Protein	#02-05p	#02-02b	#02-06p	#02-03b	#02-24p	#02-25b	#02-33p	#02-34b	#02-37p	#02-36b	#02-39p	#02-38b
5	14-3-3 related					+	+						
19	Annexin 6, Calectrin (67 kDa)	+			+	+	+	+	+	+	+	+	+
60	FK506 binding protein 4	+	+	+	+	+	+			+	+	+	+
91	Ku antigen (86 kDa)	+	+	+	+			+	+	+	+	+	+
102	Phosphoglyceromutase	+	+		+	+	+	+	+	+	+	+	+
103	Plasminogen activator inhibitor-2									+	+	+	+
104	Plasminogen activa. inhib.-2 var.									+	+	+	+
115	Rho A	*	*	+	+			+	+	+	+	+	+
121	TCHTP							*	*	+	+		
122	TCHTP var.							*	*			+	+
130	Vimentin	+	+		+	+	+	+	+	+	+	+	+

In addition, for the patients A, B and E with refractory leukemia, there were additional samples available from the time of relapse. The intervals for the date of collection from the first sample were: 6 months for patient A, 14 days for patient B, and 3 days for patient E. Analysis of the spot patterns from these samples showed an identical profile as compared to the previously collected samples of the same individual (data not shown).

**Figure 3:**
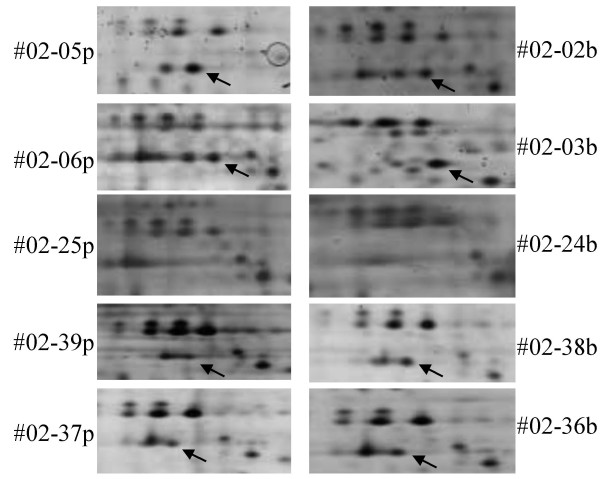
**Detail of the two-dimensional patterns with different expression of the Ku antigen**. Selective deficiency of spot 91 (Ku antigene, marked with an arrow) in patient C whereas expression is detectable in other patients irrespectively of sample origin.

Six proteins with two additional variants were found to be expressed differentially within bone marrow and peripheral blood cells of selected individuals (Table 2). Spot No. 5 (14-3-3 related) was only present in patient C, spot No. 121 and 122 (TCHTP and variant) was only present in patient F and G, respectively. Spot 60 (FK506 binding protein 4) was absent in patient D and spots No. 91 (Ku antigen) and 115 (Rho A) were not present in patient C (Figure 3). Furthermore, only patients F and G showed an expression of plasminogenactivator inhibitor-2 and a variant (spots 103 and 104).

## Discussion

Analysis of cell populations *in vivo *can provide the highest degree of fidelity for a snapshot of the protein changes that occur as a cause or consequence of the malignancy. Proteins rather than genes or mRNAs represent the key players in the cell. Expression levels of proteins determine the cellular phenotype and its plasticity in response to external signals. The aim of this study was to investigate the protein expression profiles of myelogenous blasts from patients with AML collected from two compartments, bone marrow and peripheral blood.

We previously used a cell culture model derived from thermoresistant gastric cancer to build up a database for 2D-electrophoresis patterns [17]. After matching the gel images of the AML samples with the images of the gastric cancer cell line, we found some differences in the protein patterns but overall, these changes were small: Seven proteins (with two variants) were clearly defined in the gastric carcinoma cell line but not in the AML samples (Spots-No. 4, 64, 103, 108, 114, 121, 123) (Table 3). The majority of these proteins have unspecific or unknown functions or they are clearly related to tissues and not to hematological cells [18-23].

**Table 3:** Proteins as expressed in the gastric cell line but not in AML.

*No*.	*Protein name*	*General function*	*Ref*.
4	14-3-3σ	Adapter protein for phosphoserin motifs. Regulated the interactions and subcellular localization of signalling molecules.	[18]
64	γ-Catenin	Forms a complex with α- and β-Catenin to Cadherins, that are involved in the formation and maintenance of the histo-architecture.	[19]
103	Plasmonogen activator inhibitor-2 (PAI-2)	Involved in the regulation and inhibition of binding between urokinase-type plasminogen activator and its receptor, involved in physiological and pathological proteolysis and extracellular matrix degradation.	[20]
108	Proteasome δ	Cleavage at peptide bonds with very broad specificity	[21]
114	Reticulocalbin (RCN)	RCN is a member of the EF-hand Ca(2+)-binding protein family and may regulate calcium-dependent activities in the endoplasmatic reticulum lumen or post-ER compartment	[22]
121	TCHTP	Cytoplasmatic Ca2(+)-binding protein	[23]
123	Transgelin 2	Unknown	

As an example, protein spot No. 4 (14-3-3σ) is a family member of proteins that regulate cellular activity by binding and sequestering phosphorylated proteins. 14-3-3σ promotes pre-mitotic cell-cycle arrest following DNA damage, and its expression can be controlled by the p53 tumor-suppressor gene [24]. None of the investigated AML-samples exhibited a 14-3-3σ expression in the 2D pattern. Analysis of other AML samples which did not meet the inclusion criteria for this investigation showed similar results: the expression of 14-3-3σ in AML blast is an infrequent event. This observation corresponds to investigations in breast cancer and small cell lung carcinoma. In breast cancer a hypermethylation of the CpG island of the σ gene was found that leads to gene silencing and absence of 14-3-3σ. The authors conclude, that the loss of σ expression contributes to malignant transformation by impairing the G_2 _cell cycle checkpoint function, thus allowing an accumulation of genetic defects [25,26].

Interestingly, there were only marginal differences in the expression profiles comparing patient to patient. This was also observed in studies with patients with B-cell chronic lymphocytic leukemia (CLL). In CLL, analysis allowed the identification of proteins that clearly discriminated between the patients groups with defined chromosomal characteristics or clinical parameters such as patient survival [27].

Expression of the plasminogen activator inhibitor-2 (PAI-2) was only found in patients E and F with the subtyp FAB M0 and M4, respectively. This finding is inline with data from the PAI-2 serum levels of patients with hematological malignancies, where different expression levels were correlated with different serum levels for PAI-2 in the AML subtypes FAB M4 and M0 [28]. As an explanation it was postulated, that myeloid blasts, like their non-tumoral counterparts, monocytes/macrophages, are able to synthesize most components of the plasminogen activation system. Among the numerous features shared by normal monocytes and M4 cells were the capability to migrate to areas of inflammation and to infiltrate extramedullary tissues like gingival enlargement [29].

Furthermore, we have observed that the protein patterns from samples from bone marrow and peripheral blood from the same patient show a high correlation. The observed changes are marginal and inter-individually variable.

## Conclusion

In conclusion, the protein expression profile in AML blasts collected from bone marrow aspirates in comparison to blasts from peripheral blood samples do not differ basically. This may indicate, that samples of peripheral blood with high amounts of blasts are to be considered suitable for investigations of the proteome using 2D-electrophoresis. Furthermore, protein expression profiling is likely to further impact the analysis of mechanisms involved in acute leukemia by examining routinely available biological material.

## Competing interests

The authors declare that they have no competing interests.

## Authors' contributions

GH, AL, DN, and JP carried out the 2D electrophoresis and all other experimental work. PS, ET, and WKH coordinated the laboratory work and helped to draft the manuscript. All authors read and approved the final manuscript.

## Note

^1^%T = [(acrylamide + bis-acrylamide) × 100]/total weight

%C = (bis-acrylamide × 100)/(bis-alcrylamide + acrylamide)
